# Screening for multi-drug-resistant Gram-negative bacteria: what is effective and justifiable?

**DOI:** 10.1007/s40592-020-00113-1

**Published:** 2020-04-30

**Authors:** Niels Nijsingh, Christian Munthe, Anna Lindblom, Christina Åhrén

**Affiliations:** 1 Centre for Antibiotic Resistance Research (CARe), University of Gothenburg, Gothenburg, Sweden; 2 Department of Philosophy, Linguistics and Theory of Science (FLoV), University of Gothenburg, Gothenburg, Sweden; 3 Institute for Ethics, History and Theory of Medicine, Ludwig-Maximilians University, Lessingstr. 2, 80336 Munich, Germany; 4 Department of Infectious Diseases, Institute of Biomedicine, Sahlgrenska Academy, Gothenburg, Sweden; 5 Department of Clinical Microbiology, Sahlgrenska University Hospital, Gothenburg, Sweden; 6 Swedish Strategic Program Against Antimicrobial Resistance (Strama), Region Västra Götaland, Gothenburg, Sweden

**Keywords:** Symptomatic infection, Colonisation, Antibiotic resistance, Hospital screening, Risk, Public health ethics

## Abstract

Effectiveness is a key criterion in assessing the justification of antibiotic resistance interventions. Depending on an intervention’s effectiveness, burdens and costs will be more or less justified, which is especially important for large scale population-level interventions with high running costs and pronounced risks to individuals in terms of wellbeing, integrity and autonomy. In this paper, we assess the case of routine hospital screening for multi-drug-resistant Gram-negative bacteria (MDRGN) from this perspective. Utilizing a comparison to screening programs for Methicillin-Resistant *Staphylococcus aureus* (MRSA) we argue that current screening programmes for MDRGN in low endemic settings should be reconsidered, as its effectiveness is in doubt, while general downsides to screening programs remain. To accomplish justifiable antibiotic stewardship, MDRGN screening should not be viewed as a separate measure, but rather as part of a comprehensive approach. The program should be redesigned to focus on those at risk of developing symptomatic infections with MDRGN rather than merely detecting those colonised.

## Introduction

Antibiotic resistance (ABR) is widely considered to be a threat of global proportions. Due to a combination of factors, such as overtreatment, veterinary use of antibiotics, pollution and lack of new classes of antibiotics, a situation is developing where existing treatment for currently relatively innocent infections, prophylactic use in for example surgery, and treatment of the most vulnerable patients such as neonates and immunocompromised patients is jeopardized (Miller-Petrie et al. 2017; Teillant et al. 2015; Abbo and Ariza-Heredia 2014). ABR, therefore, presents a potential threat to the everyday functioning of healthcare (WHO 2015), and healthcare institutions should make considerable effort to limit this threat. Facing such grave prospects, it thus seems that drastic action is easily justified. However, ill-advised interventions bring risks of their own (Nijsingh et al. 2020), and policies that are perceived to be unfair or otherwise weakly justified may threaten the alliance between society and the public necessary to secure the aims of public health endeavours, such as antibiotic stewardship policies (Munthe et al 2019; Munthe and Nijsingh 2019).

Most public health ethics frameworks agree that the values of effectiveness, fairness and proportionality are central to evaluating the design and implementation of specific and concrete measures, also regarding infectious disease control (Ten Have et al. 2010; Verweij 2011). However, the problem in assessing ABR interventions is that although the biological mechanism underlying resistance is quite simple, the pathways of developing ABR are notoriously complex, and it is difficult to assess what interventions are worthwhile, especially considering that different interventions may interact. In this context, the problem of ABR has been called a '(super-)wicked problem' (Littmann and Viens 2015; Rump et al. 2018), a class of complex and intractable societal challenges for which it is notoriously difficult to assess the justifiability of interventions. In this paper, we will focus on one ABR intervention, namely routine hospital screening for colonisation with multidrug resistant Gram-negative bacteria (MDRGN). In particular, we will consider multidrug resistant Enterobacterales, such as *Escherichia coli* (*E. coli*) and *Klebsiella pneumoniae*. In our discussion, we leave to one side multidrug resistant *Pseudomona*s and *Acinetobacter* species, given the relevant differences in epidemiology, microbiology, transmission, and environmental persistence (WHO 2017).

MDRGN screening is a standing policy in hospitals in Sweden and several other countries with low prevalence of these bacteria. This policy includes patients who have been hospitalized abroad the previous 6 months (ECDC 2011, 2016; CDC 2013; Wilson et al. 2016; FoHM 2017a; Public Health England 2020). We will use Sweden as a case in point, noting that arguments and conclusion are relevant for recommended and implemented hospital screening policies throughout Europe and North America. These screening programs not only include admission screening of patients hospitalised abroad but also those admitted to high-risk units such as intensive care, neonatal, haematology and transplant wards. This design has its origin in earlier screening for MRSA (Methicillin-Resistant *Staphylococcus aureus*) and VRE (Vancomycin-Resistant Enterococci), but with the emergence of MDRGN the inclusion criteria have been extended accordingly, especially for carbapenemase-producing Enterobacterales (CPE). Recently, the Swedish screening policy has been extended to recommend screening for MDRGN in patients who have travelled to high-endemic countries for more than 2 months in the past 6 months (FoHM 2017a). Similar suggestions can also be found in the literature (Van der Bij and Pitout 2012; Paltansing 2013; Lübbert et al. 2015). Also, in new guidelines from Public Heath England screening recommendations have been considerably extended and screening is no longer limited to recent hospitalisation abroad (Public Health England 2020).

In the following, we explain what screening for multi-drug resistant *colonisation* involves and how it differs from screening with an eye to the risk of symptomatic infection with multi-drug resistant *bacteria*.^1^ We also compare screening recommendations for MDRGN and MRSA in this light. We continue this comparison, assessing the effectiveness of screening in the light of known general ethical downsides of screening programs, and conclude that there are some significant differences between these pathogens, which should prompt reconsideration of the MDRGN screening policy: A fair and proportional policy with regard to MDRGN needs to focus on patient risk-factors of developing subsequent symptomatic infection with these bacteria, for instance recurrent urinary tract infections, not colonisation as such. To secure general safety, we suggest that such a justifiable ABR screening should be combined with other, well established primary prevention infection control strategies.

## Screening for multi-drug resistant bacteria

Multidrug resistant (MDR) bacteria pose a significant threat to society in general and the safe application of medical care in particular. As drug resistance rises, healthcare-associated infections become an increasing threat to medical practice, because infections caused by MDR bacteria are associated with higher incidences of mortality and prolonged hospital stay (Cassini et al. 2019; De Kraker et al. 2011). The prevention of MDR outbreaks is therefore of great importance to the proper functioning of healthcare institutions, such as hospitals and nursing homes. Healthcare institutions have various tools at their disposal to combat this threat, such as increased hygiene precautions and antibiotic stewardship (Tacconelli et al. 2014). One very powerful tool is the knowledge provided by surveillance. Specifically, screening of patients can provide information that enables institutions to take preventative action, such as contact precautions.

Sweden has been a forerunner in successful policies to manage the threat of antibiotic resistance (Mölstad et al. 2017), and has implemented an active “search and destroy” screening policy to manage this threat in healthcare.^2^ Patients at risk of being colonised with MRSA at admission to hospital have routinely been screened for colonisation since 2000. In 2007, Vancomycin-Resistant Enterococci were added to the list of reportable multidrug resistant bacteria, as well as the multidrug resistant Gram-negative bacteria (MDRGN), such as the gut bacteria *Klebsiella pneumoniae* and *E. coli* that produce Extended-Spectrum Beta-lactamases (ESBLs) and/or carbapenemases (FoHM 2017a). These bacteria are resistant to our most valuable and most used antibiotics. The guidelines for MDRGN adopt similar criteria as for MRSA. Given the seriousness of the threat, this expansion of attention is understandable: MDRGN are on the rise globally (Karanika et al. 2016; Cassini et al. 2019), which increasingly poses problems in terms of morbidity and mortality (Tacconelli et al. 2014; Trecarichi and Tumbarello 2017). Considering the lack of treatment choices for serious infections with these pathogens (Trecarichi and Tumbarello 2017), this development should be taken as a serious threat to the health of patients at special risk of MDRGN symptomatic infection.

However, as mentioned, the updated guidelines also recommend screening for additional patient groups, in particular travellers to high-endemic regions. This, too, appears to make sense at first sight. Many patients, particularly those who visit high-endemic countries, are colonised by MDRGN (Woerther et al. 2017). A large proportion of people in Sweden travel to high-endemic regions, such as South Asia, and traveller studies suggest that approximately one third are carriers of MDRGN upon return (Vading et al 2016; Woerther et al. 2017). They may therefore be considered ‘high risk’ with respect to MDRGN *colonisation*. However, we will argue that from an institutional as well as from an individual patient perspective there are reasons to treat MDRGN colonisation differently from MRSA colonisation. Using identical inclusion criteria for MDRGN screening as in the case of MRSA and expanding it to also include recent travel to high-endemic settings makes the program inadequately adjusted to the epidemiological characteristics of MDRGN, resulting in insufficient effectiveness to justify the program in light of its downsides, thus failing the public health ethical criterion of proportionality.

## Ethics and effectiveness

Screening programs are generally known to produce specific downsides such as high running costs, and risks to individuals in terms of patient autonomy, stigma and the risk of a false test result (Juth and Munthe 2012). Such downsides (to be elaborated further below) must therefore be demonstrably balanced by public health benefits to be justified. Routine hospital screening for MDR bacteria generally involves little, if any, consent, since it is a part of mandatory hospital infection control policies, illustrating that the effectiveness needed to justify the program is expected to be in terms of public health and safety.^3^ If the program is sufficiently effective in this regard, the downsides for individuals could be justified by holding them out as regrettable but necessary side-effects of a proportional policy to protect potential victims of MDR infection, and the institutions that provide care for them. Whether or not a screening program is sufficiently effective to be proportional depends, among other factors, on the treatment options, the prevalence of the condition screened for, and the specificity and sensitivity of the test (Nijsingh et al. 2017).

Public health ethics frameworks tend to place a heavy emphasis on effectiveness and proportionality (Bensimon and Upshur 2007; Childress and Bernheim 2003; ten Have et al. 2010). Effectiveness in this context is not just a cost–benefit consideration, but includes moral requirements that stem from the tension that arises when individual claims are put under pressure in the context of weighty public health concerns. Most, if not all, large scale public health interventions involve some sort of trade-off between various individual and collective interests (Holland 2007). Given the vulnerability of the individual in such contexts, public health ethical frameworks tend to emphasize the importance of thoroughly analysing and comparing alternative strategies, so that any infringement of individual claims are justified by being reasonably effective for enhancing public health without being unnecessarily intrusive or harmful.^4^ The constraint of proportionality and the weight it places on effectiveness is a safeguard against unnecessary harm and injustice, and to ensure that individual claims are not brushed aside too lightly in the face of massive threats. In addition, effectiveness is essential for justifying the opportunity costs of any large public health effort, that is, the resources spent on this effort rather than other important actions.

To determine the extent to which the screening policy with respect to MDRGN is sufficiently effective to meet the proportionality condition, it is illustrative to first consider the practice of screening for MRSA.^5^ Not least since the downsides for individual patients may be assumed to be similar (see further below). The current Swedish policy to screen for MRSA has been successful in containing the spread of hospital acquired infections, but not uncontroversial (Diekema and Climo 2008; Skyman et al. 2010; Skyman 2014; Rump 2016; Wenzel et al. 2008; Edmond et al. 2008). Before moving on to our main point, we will first give a short critical appraisal of the screening programme for MRSA and the considerations that play a role in structuring the policy.

MRSA screening is not applied universally,^6^ and universal screening is rarely advocated, because of the burdens and costs to individual patients and the difficulty of justifying the opportunity costs for the health care system as a whole (Collins et al. 2011). Rather, specific screening is performed on high-risk target groups, for example, patients with an active wound infection and patients who indicate that they have previously been admitted to a hospital with known outbreaks, or who have spent time in high-endemic settings. In case of a positive MRSA test, the patient is registered as carrier, and contact restrictions are applied. In Sweden, like in other Scandinavian countries and the Netherlands, screening programmes have been set up to minimize transmission of pathogenic bacteria and to prevent hospital outbreaks. In-hospital restrictions in case of a positive test are quite burdensome, involving isolation and intensified hygiene measures (Rump 2016; Socialstyrelsen 2010). Furthermore, upon release, patients in Sweden are bound by law to take precautions in their daily life (for instance, with respect to family members (Skyman 2014). Patients remain registered as a carrier of MRSA, until they repeatedly test negative.^7^

The negative effects of being classified as carrier, especially MRSA carriership, and the subsequent contact precautions have been extensively described (Skyman 2010, 2014; Rump 2016). MDR bacteria are scary, particularly if (as is frequently the case in public campaigns and media portrayals) advertised as ‘superbugs’, or even ‘killer bugs’. This may impress on individuals the importance of being cautious not to transmit this contagion even further. It may also fuel irrational and unnecessary fears in both patients and healthcare workers. As a consequence, patients form their own strategies to cope with the contagion limiting their social contacts in daily life and they may become overly worried, stressed and even depressed (Rump 2016; Skyman 2010, 2014). In addition, healthcare providers, family members and society as a whole may also respond to a positive test with unfounded fear and anxiety, leading to discrimination, exclusion and other stigmatizing behaviour (Wiklund et al. 2013, 2015; Skyman 2014). It has been documented that patients in contact isolation tend to receive less care than patients in general care (Saint et al. 2003; Evans et al. 2003; Stelfox et al. 2003). There are thus real and significant risks and burdens to the individual being identified as a carrier of MDR bacteria.

Although the inclusion criteria for MRSA screening aim to detect those who are (at a higher risk of) developing symptomatic infection with MRSA, they mostly serve to single out those who have become colonized not to transmit this contagion to co-patients. Usually carriers are not treated with oral antibiotics, but in MRSA carriers, attempts to decolonise patients with a chlorhexidine wash and a nasal antibiotic ointment is performed in some healthcare institutions (Henderson 2006). However, long-time carriers of MRSA may be treated with broad spectrum antibiotics to achieve decolonisation. This may benefit the patient, since it may prevent future MRSA symptomatic infections (Verbrugh 2009), but it may also pose a threat both to the patient treated, disturbing the gut flora, and to society by driving ABR development (Rottier et al. 2015). Nevertheless, on the whole, screening has been judged to be sufficiently beneficial to the healthcare institutions involved, and society in general (Vriens et al. 2002).

The MRSA screening program, although not universal, is relatively broad, as there would otherwise be substantial risk that the program misses cases (where patients do not meet inclusion criteria). This increases the running costs and the risk of burdens to individuals compared to a more restrictively targeted program. However, we agree that the importance of responding to ABR, and the effectiveness of the screening in this respect means that this measure to prevent nosocomial spread of MRSA is proportional. In the next section we will present reasons to doubt that this rationale can be extended to the current screening programs for MDRGN.

## Epidemiological comparison MRSA vs. MDRGN

Our reasons for doubting that a screening program for MDRGN similar to that for MRSA is sufficiently effective to be justified in terms of proportionality are grounded in a combination of the epidemiological characteristics and general risk management contexts of MDRGN in comparison to MRSA.

First, there are differences in the spread of the different types of resistant bacteria concerned. Contrary to MRSA, the MDRGN targeted by current screening programs typically reside in the gut, and usually do not survive very long outside the human body. The risk of nosocomial spread due to colonisation therefore appears to be rather low in the absence of diarrhoea (Hilty et al. 2012). This is true in particular of MDR* E. coli*, which is by far the most dominant MDRGN.^8^ Contrary to for example staphylococci and enterococci, most MDRGN require moist environments to survive for a longer period of time outside the human body (Hirai 1991). In addition, flakes of skin from MRSA colonised individuals present a high risk of nosocomial spread, as does nasal colonisation in combination with sneezing. Also, MRSA skin colonisation involves significant risk of indirect spread via the hands of healthcare professionals if not properly disinfected before and after patient care (Siegel et al. 2007).^9^ Since transmission of MRDGN, especially for *E. coli*, primarily occurs through the fecal–oral route, the risk of nosocomial spread is much smaller—although there are exceptions to this rule, for example when the patient has diarrhoea or when there are other risk factors involved that increase the risk of indirect spread of MDRGN from the gut flora, such as urinary catheters and gut stoma. We will return to these exceptions, and their relevance to high-risk units, later.

Second, identification of an MRSA colonised patient in a hospital is comparatively more important from a risk management perspective. The MRSA colonisation rate in the general community is rather low in Europe (Mölstad et al. 2017). As consequence, when a colonised person enters a hospital, where they will come into close contact with others (some of whom especially vulnerable), this marks a significant increase of the risk of transmitting MRSA. In contrast, MDRGN colonisation occurs mostly via contaminated food and water, and the rate of community colonisation for MDRGN is therefore much higher and steadily increasing, even in low endemic settings (Woerther et al. 2013; Karanika et al. 2016). This means that there will already be a relatively large number of undetected cases of MDRGN in the hospital, thereby reducing the risk management significance of identifying a few more. Of course, making the screening program universal may address this particular aspect, but as already explained, this would at the same time add substantial downsides in terms of opportunity cost and individual burdens.

A third difference between MRSA and MRDGN concerns the relationship between colonization and symptomatic infectious disease. Carriers of MRSA are known to have an increased risk of subsequent symtomatic infection (Van Belkum and Verbrugh 2001; Nelson et al. 2018), whereas the risk of subsequent symtomatic infection in gut carriers of MDRGN is largely unkown (Turbett and Mansour 2016; Rottier et al. 2015; Goulenok et al. 2013; Boldt et al. 2018). Recent reports indicate a very low frequency of subsequent symtomatic infection in a large unselected patient group of gut carriers (Lindblom et al. 2018; Boldt et al. 2018).^10^ This is particularly salient since identification of an MDR carrier status increases the risk of broad-spectrum antibiotic overuse, which may have negative consequences for both individual and society. Specifically, it increases the likelihood that carbapenems and other drugs of 'last resort'are used for empirical treatment of carriers of MDRGN (Papp-Wallace et al. 2011; Vardakas et al. 2012) in case of a symptomatic infection in these patients until clinical culture results are available determining the actual cause of disease. In addition, there is evidence that most MDRGN strains found by faecal screening are less virulent and thus less likely to cause symptomatic disease (Ny et al. 2016).

This relates to the next point. The colonisation status in MRSA is 'treatable', in contrast to MDRGN, where attempts to eliminate the carrier status by decolonisation strategies have been disappointing (Turbett and Mansour 2016). In short, while identifying a MRSA carrier may help to prevent infection in addition to secondary transmission and hospital outbreaks, and to eventually relieve the patient of the colonisation, the identified MDRGN carrier cannot be subjected to either enhanced effective infection control unless they have risk factors contributing to spread like diarrhoea or to treatments that may decolonise the patient. The only action on offer would be contact precautions and isolation that are difficult to motivate either ethically or in medical terms, other than in high risk units like haematology or ICUs, or empirical treatment with last resort antibiotics, which would be unsustainable and unjustifiable from an antibiotic stewardship standpoint. As a consequence, the dilemma is thus between increasing burdens on patients and institutions in a (rather imperfect) attempt to prevent further spread, or accepting the ineffectiveness of the screening programme.

Finally, setting up a screening program for MDRGN that would have a chance of having some infection prevention effect is complicated by the fact that MDRGN resistance is mostly plasmid mediated and several different species are involved. This means that resistance traits can be transferred from individual bacteria to other individual bacteria, and in rare cases even between species (Bosch et al. 2017; Lindblom et al. 2019). Consequently, to the extent that individuals develop symptoms by resistant bacteria due to an MDRGN colonisation, these may not be the bacteria targeted by screening, but rather from *other* bacteria in their gut adopting the same plasmid (Hagel et al. 2019). A potentially effective program would therefore have to focus not on particular drug resistant bacteria types, but also on the resistant mechanism for early identification of polyclonal outbreaks that may involve interspecies plasmid migration (Müller et al. 2016). This complication does not apply in the same way to MRSA, since only one species is involved and the resistance mechanism in MRSA is chromosomal (Deurenberg et al. 2007; see however Becker et al. 2018).

Summarizing, screening for MDRGN in comparison with the current programs of MRSA screening will be less effective in at least four ways: (1) secondary transmission from colonised patients without risk factors for transmission like diarrhoea is less likely especially in the case of the most common species (*E. coli*), (2) these programs find a smaller proportion of the colonised population since the community reservoir is becoming increasingly much larger for MDRGN than MRSA, (3) a larger proportion of the identified patients will likely not get sick and an ethically justifiable or public health sustainable intervention to reduce infection seems out of reach, and (4) such programs present difficulties in targeting the right strain since several different bacteria and resistance mechanisms are involved.

Taken together, these observations put into question the justifiability of the MDRGN screening regimes currently implemented in several countries, such as Sweden and the Netherlands, as these programmes still have the same ethical and economical downsides as their MRSA siblings (Wiklund et al. 2013), but lack the effectiveness of the latter. This undermines both the proportionality and the justification of the opportunity costs of the program. A positive diagnosis of MDRGN colonisation will have a significant negative impact on people’s lives, with no or very little benefit to either the tested person or to public health. As colonisation of MDRGN bacteria increases even more,^11^ the screening will involve more and more people, where each positive case threatens to provide a negative balance of the reasons that might otherwise have supported the programme. In short, more people are burdened and even harmed and higher costs are accumulated with weaker justification than in the case of MRSA.

None of this is to downplay the seriousness of MDRGN, in general and as a danger to health care institutions such as hospitals. Also, the arguments presented so far are relative to the case for screening for MRSA—the fact that screening for MDRGN is less effective than screening for MRSA does not undermine all conceivable types MDRGN hospital screening efforts. However, the apparently weak prospect of a sufficient justification for the current MDRGN screening policy provides good reason to at least look for better options. In the next section, we sketch an alternative proposal to this effect.

## Alternatives to the current programme

An alternative to the wide net–small mesh approach copy-pasted from MRSA colonisation screening programs, the differences between MRSA and MRDGN would suggest a strategy that change focus from merely detecting colonisation to a more specific focus on those groups that are at particular risk of developing symptomatic infection, or at specific risk of serious harm in case of MDRGN infection, or where infection brings a significant risk of spread. More precisely, the focus should be on foreseeing symptomatic infection, preventing outbreak, and preventing serious consequences for patients due to infections. This implies that screening for MDRGN should not be viewed as an isolated policy, but rather as one part of a comprehensive approach that includes both general and specific primary and secondary prevention strategies.

The first action to highlight from that perspective is not screening, but general primary prevention. This is important, as it links to the mentioned opportunity costs: ineffective screening programs will use resources that could instead be applied to ensure that there is less of importance for screening programs to capture in the first place. Hospitals and other health care institutions could further enhance measures that curb all healthcare associated infections: high compliance to basic hygiene routines in all care, good toilet and food hygiene, single rooms with toilet to everyone in hospital and so on (ECDC 2016; Lemmen and Lewalter 2018). None of these measures are of course specific to the approach to MDRGN. Yet, in the context of assessing whether screening is worthwhile and in what form, they deserve specific mention, because they take the focus from finding instances of colonization to preventing infection and transmission of all potential pathogens. This leaves the question, what, in this broader context, would be the added value of screening or testing for colonization? We submit that there may be reasons to screen patients in some, specific circumstances, in order to apply specific primary and secondary prevention measures.

Although most MRDGN carriers will not get sick or give rise to dangerous spread, empirical treatment in patients with an elevated risk of MDRGN *symptomatic infection* is lifesaving, and ABR is an independent predictor of mortality (Wilson et al. 2016; Goulenok et al. 2013; Rottier et al. 2015; Turbett and Mansour 2016). In these cases, both the patient and the treating physician will benefit from knowing about a possible carrier status in case of subsequent infection episodes. Moreover, patients with symptomatic infection generally are much more contagious than carriers, for the simple reason that there are more bacteria involved, creating a straightforward infection control reason in support of this focus.

One straightforward way to target this group is to simply keep track of those already diagnosed with such an infection. Recurrent infection is a well-known phenomenon in patients with urinary tract infections (UTI) due to sensitive *E. coli* and have been reported in approximately 30% of those with a prior UTI or septicaemia due to ESBL-producing MDRGN, primarily MDR *E. coli* (Lindblom et al. 2018). In addition, patients with known risk factors for developing disease with MDRGN should be screened, such as antibiotic treatment within the last months, urological comorbidity, immunosuppression, use of urinary catheters, and those who have had a previous MDRGN infection.^12^

Additionally, there are specific circumstances where the risk of spread in case of infection,^13^ or the seriousness of the consequences of such a spread (haematology, transplant and neonatal units, ICUs), creates an immediate infection control reason to screen for MRDGN colonisation. Discovering MDRGN colonisation in these contexts in order to apply contact precautions or clinical treatment measures in case of serious risk would be in the general interest of all patients concerned, and also well motivated from a general public health standpoint.

Given that universal screening for MDRGN (or MRSA)—where all people in a population, or institution, are screened—is unjustified, a screening program that may effectively add to important infection control against a background of sound general infection control routines, can be motivated on the basis of proportionality and fairness. More specifically, we propose that the current MRDGN screening programs should be redesigned to target patients:

1. Who are at particular risk of developing MDR disease due to MDRGN colonisation, for instance patients with a history of recent antibiotic treatment, previous infection with MDRGN bacteria, urological disorders etc.
2. Who have been directly transferred for further hospitalisation from a hospital in a high endemic area,
3. Hospitalized in units where a possible infection would bring significant risks of spread and outbreak, and
4. Hospitalized in units where spread will have catastrophic results for all patients (including the screened individuals) due to their high risk of developing and vulnerability to infections.^14^

To further increase effectiveness (and reduce ethical downsides), the management of positive results in the program should also be reformed. Specifically, healthcare providers should be properly informed about MDR bacteria, the risk of spread and how to protect themselves and the patients from becoming colonized. Positively tested patients should receive the same level of care as others and communication should be focused on eliminating stigma. Note that none of these measures are likely to be very effective unless there is sufficient knowledge among healthcare professionals and the public in general on bacterial infections, resistance and the purpose of screening. Therefore, any effective approach should be accompanied by active information and education efforts. Also, since it is not immediately obvious that the individual should pay the costs of the consequences of a positive test, another action worthy of serious consideration is to apply compensation schemes to those harmed or disadvantaged by restrictions (Rump et al. 2018).

We repeat the importance of the premise that such a screening program assumes enhanced efforts to apply hospital infection control across the board. Measures that curb all health-related infections, high appliance to basic hygiene routines in all care, good toilet and food hygiene, single rooms with toilet to everyone in hospital and so on (ECDC 2016) will also curb the threat of MRDGN and reduce the need for unnecessarily burdening and costly screening programs.

## Concluding discussion

We have argued that current routine hospital screening efforts for MDRGN colonisation are relatively ineffective, and that policy should be reformed to emphasize the aim of reducing symptomatic MDRGN infection and hospital outbreaks, and to apply adequate treatment and infection control measures to this effect. Our proposal mixes a higher emphasis on general primary prevention strategies in hospitals, a more focused screening program for MDRGN colonisation, and more measured actions in case of positive test results that may address known risk of stigma and unnecessary intrusion for patients. This reform would address both the justification of the opportunity costs of the policy, its ethical downsides, and its overall prospect of justification.^15^

Screening is ethically complicated due to the, often considerable, general costs and ethical downsides of screening programmes (Juth and Munthe 2012). Screening for infection control reasons tends to add additional stress on this complexity, as there is often no clear prospect of benefit at all to positively tested individuals (Nijsingh et al. 2017). But the ABR context is different from other public health and infection control dilemmas in several respects (Rump et al. 2018). This affects the ethical analysis of screening for the aim of antibiotic stewardship. ABR has been described as a slowly emerging crisis or disaster for health systems and healthcare (Viens and Littmann 2015), and will certainly affect healthcare institutions and many patients in profound ways. It is also a chronic problem, in the sense that there is not likely to be a quick solution and societies will have to find ways to adapt to this challenge. This calls not only for swift action to curb serious threats at the population and institutional levels, but also for sustainable policy that is well justified from an ethical standpoint, and that can inspire necessary political and popular legitimacy. Our analysis of current routine hospital screening programs for MDRGN colonisation illustrates the importance of not being lured into rash measures by the massive stakes of the ABR challenge, but to consider the actual effectiveness of each step in proposed policies in light of their (inescapable) ethical downsides and pragmatic risks.

Our argument illustrates that the consideration of such effectiveness is not limited to simple trade-offs between individual and collective interests of the sort often highlighted in public health and screening ethics. Rather, in the ABR context, adequately designed screening programmes can be part of what it means for a hospital (and health system) to deliver good care, both for the patient who is screened and those who are at risk of actual MDR disease. We have argued that, in the case of MDRGN colonisation, a more measured and focused hospital policy would also be easier to justify in this way.

There remains in our proposal the ethical downside of screening without collection of informed consent. In some public health ethical recommendations, such as the so-called intervention ladder of the Nuffield Council of Bioethics (Nuffield 2007), providing choice for concerned parties is a highly prioritized aspect of public health interventions, and severely restricting choice, or eliminating it altogether, is viewed as last resort option when stakes are extreme. We agree with Dawson (2016) that this one-dimensional way of “prioritizing liberty” fails to capture the main aims and values of public health policy and action, and how they interplay with other ethical considerations. While we do agree that considerations of individual liberty should play a part in the justification of public health policy and action, it should not be given a rigid superior priority relative to all other values, but considered in proportion to the way in which public health actions many times serve to protect the opportunities of all people by furthering general health goals and institutions of structural importance (Munthe 2008). While individual rights have a central place in a public health ethical framework, these need to consider the role of population health and linked social institutions to facilitate the effective discharging everyone’s equal rights across time. If ABR is not controlled and managed well, it threatens to undermine the entire system of effective modern healthcare within which the patient’s right to choose is supposed to be operating. For similar reasons, a principle of subsidiarity (urging that power over decisions should not be unnecessarily centralized) would not speak against well-balanced screening programs for ABR colonization in hospitals (Kotalik 2010).

The argument we have developed for our proposed reform of hospital MDRGN prevention policy, including screening focused on prevention and treatment of actual disease and outbreak, illustrates this. Lack of choice can be motivated from an ethical standpoint if sufficient antibiotic stewardship effectiveness is ensured, considering scientific, ethical and pragmatic aspects. Adding procedures to preserve some sort of token recognition of individual liberty (such as information about the option to leave the hospital rather than take the test) would not be meaningful from a practical or theoretical perspective, not even from a perspective of individual rights.

## Footnotes

1 In this paper we distinguish between colonisation on the one hand, and symptomatic infection on the other. Colonisation refers to a state where the person infected by a pathogen does not display symptoms, whereas symptomatic infection refers to the state where the state of the infection causes discernable symptoms (i.e. damage) to the person. We will sometimes use’carrier state’ as synonymous to colonisation and’symptomatic infection’ for’infectious disease’. Where nothing hinges on the difference, we sometimes use just’infection’. See Casadevall and Pirofski (1999) for a more thorough discussion of the relevant terminology.

2 Another example of a very pro-active, and on the face of it successful, search and destroy policy is in the Netherlands (Wertheim et al. 2004; Bootsma et al. 2006).

3 This makes the ethical assessment of hospital screening for MDRGN colonization different from the assessment of screening programs where there are potential benefits to individual patients (such as newborn screening programs), or when the aim of the program is to enhance patient decision-making, such as prenatal screening programs (Juth and Munthe 2012, ch. 2–3), and where communicable disease management measures are not among the options. While a comparative ethical analysis with such programs would be interesting indeed, in this article we are focusing on the ethical evaluation of MDRGN colonization hospital screening in its own right. We do, however, expand on some general public health ethical considerations with regard to the limited room for informed consent and voluntariness in the kind of screening under consideration.

4 There is some technical debate regarding how the condition of unnecessary intrusion and harm should be read. While Childress and Bernheim (2003) formulate a strict necessary condition of minimal intrusion, Grill and Dawson (2017) have argued for a more balanced approach, where the resulting framework does not give any special priority to the value of liberty and comparison of alternatives involves taking into account all relevant values. However, this debate will not affect the soundness or validity of the argument pursued in the present paper, since our focus is on effectiveness, not an application of the harm principle, or the least intrusion principle. See "Concluding discussion".

5 It should be noted that the case for VRE, which is also a screening target in Sweden, is slightly different from that for MRSA. VRE is mostly a gut-coloniser, which can be found causing urinary tract infections, gallbladder infections and endocarditis—but they are rare pathogens and particularly dangerous to weak and immunocompromised patients. In most of what follows, we leave discussion of this to one side for reasons of simplicity—but note that if indeed different considerations apply to VRE, this example further supports our argument for differentiation.

6 Universal screening in this context would involve testing every (new) patient in hospital for MDR colonization.

7 In Sweden, three times over a period of 1 year (Socialstyrelsen 2010).

8 Comprising about 90% of all cases in Sweden (FoHM 2017b).

9 Of course, MDRGN can be indirectly transmitted via the hands too if the patients have risk factors for transmitting MDRGN. However, skin colonization is much more rare for MDRGN than for MRSA.

10 Tischendorf et al. (2016) argue that, in case of colonisation carbapenem resistant enterobactericeae, there is a relatively high proportion of subsequent infection. However, this study encompasses a relatively high number of (severely) ill patients in high-risk units, who are at a higher risk of developing infection per se. Nonetheless, more research is necessary to establish the risk for different resistant strains in different contexts.

11 In Sweden, at least 5% of the population is currently colonised by MDRGN (Ny et al. 2016; Mölstad et al. 2017; Lindblom et al. 2018), but given that some other countries are up to 60% (Woerther et al. 2013), it seems fair to suppose that the numbers will soon rise well above this already high proportion. By contrast, the prevalence of asymptomatic MRSA in the community is estimated to be between 1 and 2% (Mölstad et al. 2017; Den Heijer 2013).

12 The latter was found to be a strong predictor in patients with sepsis due to MDR* E. coli* (Fröding et al. 2019).

13 As mentioned earlier, this applies in particular to high-risk units, where colonization may easily spread. This is the case where, for example, catheters, stoma, tracheostoma and drains are used.

14 Note that this focus on the risk of developing symptomatic infection does not preclude, but rather necessitates, the advancement of knowledge concerning the risk of subsequent infection in MDRGN carriers in general and selected patient populations. (Lindblom et al. 2018; Turbett and Mansour 2016). There is also a need to relate the risk to the different species within the category MDRGN, knowing that some of these species are more dangerous or more likely to spread than others in a hospital setting.

15 Of course, the proof of the eating is in the pudding. Any screening programme will need continuous monitoring in order to verify its effectiveness. We do not claim that our approach has no downsides of its own or even that it is superior to all alternatives regardless of context. However, there seem to be strong reasons, based in empirical observations, to prefer our alternative to the current programmes. Future research will need to provide more evidence on for example the risk of colonisation leading to either symptomatic infection, or transmission, the possibility of successful and safe treatment of colonised individuals and value of more comprehensive testing as MDR bacteria become more prevalent.

## References

[CR1] Abbo LM, Ariza-Heredia EJ (2014). Antimicrobial stewardship in immunocompromised hosts. Infectious Disease Clinics.

[CR2] Becker K, van Alen S, Idelevich EA, Schleimer N, Seggewiß J, Mellmann A, Peters G (2018). Plasmid-encoded transferable mecB-mediated methicillin resistance in *Staphylococcus aureus*. Emerging Infectious Diseases.

[CR3] Bensimon CM, Upshur RE (2007). Evidence and effectiveness in decisionmaking for quarantine. American Journal of Public Health.

[CR4] Boldt AC, Schwab F, Rohde AM, Kola A, Bui MT, Märtin N, Zweigner J (2018). Admission prevalence of colonization with third-generation cephalosporin-resistant Enterobacteriaceae and subsequent infection rates in a German university hospital. PLoS ONE.

[CR5] Bootsma MCJ, Diekmann O, Bonten MJ (2006). Controlling methicillin-resistant *Staphylococcus aureus*: Quantifying the effects of interventions and rapid diagnostic testing. Proceedings of the National Academy of Sciences.

[CR6] Bosch T, Lutgens SP, Hermans MH, Wever PC, Schneeberger PM, Renders NH, Witteveen S (2017). Outbreak of NDM-1-producing *Klebsiella pneumoniae* in a Dutch hospital, with interspecies transfer of the resistance plasmid and unexpected occurrence in unrelated health care centers. Journal of Clinical Microbiology.

[CR7] Casadevall A, Pirofski L (1999). Host-pathogen interactions: redefining the basic concepts of virulence and pathogenicity. Infection and Immunity.

[CR8] Cassini A, Högberg LD, Plachouras D, Quattrocchi A, Hoxha A, Simonsen GS, Ouakrim DA (2019). Attributable deaths and disability-adjusted life-years caused by infections with antibiotic-resistant bacteria in the EU and the European Economic Area in 2015: A population-level modelling analysis. The Lancet Infectious Diseases.

[CR9] CDC. 2013. New carbapenem-resistant Enterobacteriaceae warrant additional action by healthcare providers; February 14, 2013. https://stacks.cdc.gov/view/cdc/25250. Accesssed 28 June 2019.

[CR10] Childress JF, Bernheim RG (2003). Beyond the liberal and communitarian impasse: A framework and vision for public health. Florida Law Review.

[CR11] Collins J, Raza M, Ford M, Hall L, Brydon S, Gould FK (2011). Review of a three-year meticillin-resistant *Staphylococcus aureus* screening programme. Journal of Hospital Infection.

[CR12] Dawson AJ (2016). Snakes and ladders: state interventions and the place of liberty in public health policy. Journal of Medical Ethics.

[CR13] de Kraker MEA, Davey PG, Grundmann H, on behalf of the BURDEN study group (2011). Mortality and hospital stay associated with resistant *Staphylococcus aureus* and *Escherichia coli* Bacteremia: estimating the burden of antibiotic resistance in Europe. PLoS Med.

[CR14] den Heijer CDJ (2013). Prevalence and resistance of commensal *Staphylococcus aureus*, including meticillin-resistant *S. aureus*, in nine European countries: A cross-sectional study. The Lancet Infectious Diseases.

[CR15] Deurenberg RH, Vink C, Kalenic S, Friedrich AW, Bruggeman CA, Stobberingh EE (2007). The molecular evolution of methicillin-resistant *Staphylococcus aureus*. Clinical Microbiology and Infection.

[CR16] Diekema DJ, Climo M (2008). Preventing MRSA infections: finding it is not enough. JAMA.

[CR17] ECDC (European Centre for Disease Prevention and Control). 2011. Risk assessment on the spread of carbapenemase-producing Enterobacteriaceae (CPE) through patient transfer between healthcare facilities, with special emphasis on cross-border transfer. https://ecdc.europa.eu/en/publications-data/risk-assessment-spread-carbapenemase-producing-enterobacteriaceae-cpe-through. Accessed 29 June 2019.

[CR18] ECDC (European Centre for Disease Prevention and Control). 2016. Last-line antibiotics are failing:options to address this urgent threat to patients and healthcare systems. https://ecdc.europa.eu/sites/portal/files/media/en/publications/Publications/antibiotic-resistance-policy-briefing.pdf. Accessed 28 June 2019.

[CR19] Edmond M, Lyckholm L, Diekema D (2008). Ethical implications of active surveillance cultures and contact precautions for controlling multidrug resistant organisms in the hospital setting. Public Health Ethics.

[CR20] Evans HL, Shaffer MM, Hughes MG, Smith RL, Chong TW, Raymond DP, Sawyer RG (2003). Contact isolation in surgical patients: a barrier to care?. Surgery.

[CR21] FoHM (Folkhälsomyndigheten). 2017a. Screening för antibiotikaresistenta bakterier. https://www.motesplatssocialhallbarhet.se/contentassets/8f56681b343b46b9a48f13c0b1774e82/screening-resistenta-bakterier-02307-2017.pdf. Accessed 28 June 2019.

[CR22] FoHM (Folkhälsomyndigheten). 2017b. Extended Spectrum Betalactamase (ESBL)-producerande Enterobacteriaceae 2017. https://www.folkhalsomyndigheten.se/folkhalsorapportering-statistik/statistikdatabaser-och-visualisering/sjukdomsstatistik/extended-spectrum-beta-lactamase-esbl/kommentarer-och-specialstatistik/2017/. Accessed 28 June 2019.

[CR23] Fröding Inga (2019). Prediction of bloodstream infection caused by extended-spectrum β-lactamase-producing Enterobacterales in patients with suspected community-onset sepsis. International Journal of Antimicrobial Agents.

[CR24] Goulenok T, Ferroni A, Bille E, Lécuyer H, Join-Lambert O, Descamps P, Zahar JR (2013). Risk factors for developing ESBL *E. coli*: Can clinicians predict infection in patients with prior colonization?. Journal of Hospital Infection.

[CR25] Grill K, Dawson A (2017). Ethical frameworks in public health decision-making: Defending a value-based and pluralist approach. Health Care Analysis.

[CR26] Hagel S, Makarewicz O, Hartung A, Weiß D, Stein C, Brandt C, Pletz MW (2019). ESBL colonization and acquisition in a hospital population: the molecular epidemiology and transmission of resistance genes. PLoS ONE.

[CR27] Henderson DK (2006). Managing methicillin-resistant Staphylococci: a paradigm for preventing nosocomial transmission of resistant organisms. The American Journal of Medicine.

[CR28] Hilty M, Betsch BY, Bögli-Stuber K, Heiniger N, Stadler M, Küffer M, Droz S (2012). Transmission dynamics of extended-spectrum β-lactamase–producing Enterobacteriaceae in the tertiary care hospital and the household setting. Clinical Infectious Diseases.

[CR29] Hirai Y (1991). Survival of bacteria under dry conditions; From a viewpoint of nosocomial infection. Journal of Hospital Infection.

[CR30] Holland S (2007). Public health ethics.

[CR31] Juth N, Munthe C (2012). The ethics of screening in health care and medicine: Serving society or serving the patient?.

[CR32] Karanika S, Karantanos T, Arvanitis M, Grigoras C, Mylonakis E (2016). Fecal colonization with extended-spectrum beta-lactamase–Producing Enterobacteriaceae and risk factors among healthy individuals: a systematic review and metaanalysis. Reviews of Infectious Diseases.

[CR33] Kotalik J (2010). Examining the suitability of the principle of subsidiarity for bioethics. Kennedy Institute of Ethics Journal.

[CR34] Lemmen SW, Lewalter K (2018). Antibiotic stewardship and horizontal infection control are more effective than screening, isolation and eradication. Infection.

[CR35] Lindblom A, Karami N, Magnusson T, Åhrén C (2018). Subsequent infection with extended-spectrum β-lactamase-producing Enterobacteriaceae in patients with prior infection or fecal colonization. European Journal of Clinical Microbiology & Infectious Diseases.

[CR36] Lindblom A, Sriram KK, Müller V, Öz R, Sandström H, Åhrén C, Karami N (2019). Interspecies plasmid transfer appears rare in sequential infections with extended-spectrum β-lactamase (ESBL)-producing Enterobacteriaceae. Diagnostic Microbiology and Infectious Disease.

[CR37] Littmann J, Viens AM (2015). The ethical significance of antimicrobial resistance. Public Health Ethics.

[CR38] Lübbert C, Straube L, Stein C, Makarewicz O, Schubert S, Mössner J, Rodloff AC (2015). Colonization with extended-spectrum beta-lactamase-producing and carbapenemase-producing Enterobacteriaceae in international travelers returning to Germany. International Journal of Medical Microbiology.

[CR39] Miller-Petrie M, Pant S, Laxminarayan R (2017). Drug-resistant infections. Major infectious diseases.

[CR40] Mölstad S, Löfmark S, Carlin K, Erntell M, Aspevall O, Blad L, Skoog G (2017). Lessons learnt during 20 years of the Swedish strategic programme against antibiotic resistance. Bulletin of the World Health Organization.

[CR41] Müller V, Karami N, Nyberg LK, Pichler C, Torche Pedreschi PC, Quaderi S, Westerlund F (2016). Rapid tracing of resistance plasmids in a nosocomial outbreak using optical DNA mapping. ACS Infectious Diseases.

[CR42] Munthe C (2008). The goals of public health: an integrated, multidimensional model. Public Health Ethics.

[CR43] Munthe C, Nijsingh N (2019). Cutting red tape to manage public health threats: an ethical dilemma of expediting antibiotic drug innovation. Bioethics.

[CR44] Munthe C, Nijsingh N, de Fine Licht K, Joakim Larsson DG (2019). Health-related research ethics and social value: antibiotic resistance intervention research and pragmatic risks. Bioethics.

[CR45] Nelson RE, Evans ME, Simbartl L, Jones M, Samore MH, Kralovic SM, Rubin MA (2018). Methicillin-resistant *Staphylococcus aureus* colonization and pre-and post-hospital discharge infection risk. Clinical Infectious Diseases.

[CR46] Nijsingh, N., N. Juth, and C. Munthe. 2017. The ethics of screening. In: *International encyclopedia of public health*. Amsterdam: Elsevier.

[CR47] Nijsingh N, Larsson J, de Fine Persson, Licht K, Munthe C, Jamrozik E, Selgelid MJ (2020). Justifying antibiotic resistance interventions: uncertainty, precaution and ethics. Ethics and drug-resistant infections: collective responsibility for global public health. Public health ethics analysis.

[CR48] Nuffield Council of Bioethics. 2007. Public health: ethical issues. London: Nuffield Council of Bioethics. https://nuffieldbioethics.org/wp-content/uploads/2014/07/Public-health-ethical-issues.pdf. Accessed 28 June 2019.

[CR49] Ny S, Löfmark S, Börjesson S, Englund S, Ringman M, Bergström J, Byfors S (2016). Community carriage of ESBL-producing *Escherichia coli* is associated with strains of low pathogenicity: a Swedish nationwide study. Journal of Antimicrobial Chemotherapy.

[CR50] Paltansing S, Vlot JA, Kraakman ME, Mesman R, Bruijning ML, Bernards AT, Veldkamp KE (2013). Extended-spectrum β-Lactamase–producing Enterobacteriaceae among travelers from the Netherlands. Emerging Infectious Diseases.

[CR51] Papp-Wallace KM, Endimiani A, Taracila MA, Bonomo RA (2011). Carbapenems: past, present, and future. Antimicrobial Agents and Chemotherapy.

[CR52] Public Health England. 2020. Framework of actions to contain carbapenemase-producing Enterobacterales. London: Public Health England. Available at: https://assets.publishing.service.gov.uk/government/uploads/system/uploads/attachment_data/file/856297/Framework_of_actions_to_contain_CPE.pdf. Accessed 29 Apr 2020.

[CR53] Rottier WC, Bamberg YR, Dorigo-Zetsma JW, van der Linden PD, Ammerlaan HS, Bonten MJ (2015). Predictive value of prior colonization and antibiotic use for third-generation cephalosporin-resistant Enterobacteriaceae bacteremia in patients with sepsis. Clinical Infectious Diseases.

[CR54] Rump, B., M.G. De Boer, R. Reis, M. Wassenberg, and J. Van Steenbergen. 2016, December. Signs of stigma and poor mental health among carriers of methicillin-resistant *Staphylococcus aureus*. In: *Open forum infectious diseases* (Vol. 3, No. suppl_1). Oxford University Press.

[CR55] Rump B, Timen A, Hulscher M, Verweij M (2018). Ethics of infection control measures for carriers of antimicrobial drug–resistant organisms. Emerging Infectious Diseases.

[CR56] Saint S, Higgins LA, Nallamothu BK, Chenoweth C (2003). Do physicians examine patients in contact isolation less frequently? A brief report. American Journal of Infection Control.

[CR57] Siegel JD, Rhinehart E, Jackson M, Chiarello L (2007). 2007 Guideline for isolation precautions preventing transmission of infectious agents in healthcare settings. American Journal of Infection Control.

[CR58] Skyman, E. 2014. *Consequences of meticillin-resistant Staphylococcus aureus (MRSA) acquisition* (Doctoral dissertation, Doctoral thesis, Gothenburg Studies in Infectious Diseases). Göteborg: Ineko AB. Tillgänglig : https://gupea.ub.gu.se/bitstream/2077/36758/1/gupea_2077_36758_1.pdf.

[CR59] Skyman E, Sjöström HT, Hellström L (2010). Patients’ experiences of being infected with MRSA at a hospital and subsequently source isolated. Scandinavian Journal of Caring Sciences.

[CR60] Socialstyrelsen. 2010. Meticillinresistent *Staphylococcus aureus* (MRSA). Rekommendationer för bedömning av bärarskap och smittrisk. https://www.lul.se/Global/Extran%C3%A4t/V%C3%A5rdgivare/Smittskydd/Dokument/Sjukdomar%20enl.%20SmL/mrsa_rekommendationer_for_bedomning_av_bararskap_och_smittrisk_2010_6_19.pdf. Accessed 9 July 2019.

[CR61] Stelfox HT, Bates DW, Redelmeier DA (2003). Safety of patients isolated for infection control. JAMA.

[CR62] Tacconelli E, Cataldo MA, Dancer SJ, De Angelis G, Falcone M, Frank U, Singh N (2014). ESCMID guidelines for the management of the infection control measures to reduce transmission of multidrug-resistant Gram-negative bacteria in hospitalized patients. Clinical Microbiology and Infection.

[CR63] Teillant A, Gandra S, Barter D, Morgan DJ, Laxminarayan R (2015). Potential burden of antibiotic resistance on surgery and cancer chemotherapy antibiotic prophylaxis in the USA: A literature review and modelling study. The Lancet Infectious Diseases.

[CR64] ten Have M, de Beaufort ID, Mackenbach JP, van der Heide A (2010). An overview of ethical frameworks in public health: Can they be supportive in the evaluation of programs to prevent overweight?. BMC Public Health.

[CR65] Tischendorf J, de Avila RA, Nasia S (2016). Risk of infection following colonization with carbapenem-resistant Enterobactericeae: A systematic review. American Journal of Infection Control.

[CR66] Trecarichi EM, Tumbarello M (2017). Therapeutic options for carbapenem-resistant Enterobacteriaceae infections. Virulence.

[CR67] Turbett SE, Mansour MK (2016). Editorial commentary: Fecal ESBL screening: Are we ready for this information?. Clinical Infectious Diseases.

[CR68] Vading M, Kabir MH, Kalin M, Iversen A, Wiklund S, Naucler P, Giske CG (2016). Frequent acquisition of low-virulence strains of ESBL-producing *Escherichia coli* in travellers. Journal of Antimicrobial Chemotherapy.

[CR69] van Belkum, A., and H. Verbrugh. 2001. 40 years of methicillin resistant *Staphylococcus aureus*: MRSA is here to stay—But it can be controlled. *BMJ* 323: 644. 10.1136/bmj.323.7314.644 PMC1121221 11566814

[CR70] van der Bij AK, Pitout JD (2012). The role of international travel in the worldwide spread of multiresistant Enterobacteriaceae. Journal of Antimicrobial Chemotherapy.

[CR71] Vardakas KZ, Tansarli GS, Rafailidis PI, Falagas ME (2012). Carbapenems versus alternative antibiotics for the treatment of bacteraemia due to Enterobacteriaceae producing extended-spectrum β-lactamases: A systematic review and meta-analysis. Journal of Antimicrobial Chemotherapy.

[CR72] Verbrugh HA (2009). Colonization with *Staphylococcus aureus* and the role of colonization in causing infection. Staphylocci in Human Disease.

[CR73] Verweij M (2011). Infectious disease control. Public health ethics: Key concepts and issues in policy and practice.

[CR74] Viens AM, Littmann J (2015). Is antimicrobial resistance a slowly emerging disaster?. Public Health Ethics.

[CR75] Vriens M, Blok H, Fluit A, Troelstravan der Werken AC, Verhoef J (2002). Costs associated with a strict policy to eradicate methicillin-resistant *Staphylococcus aureus* in a Dutch University Medical Center: a 10-year survey. European Journal of Clinical Microbiology and Infectious Diseases.

[CR76] Wenzel RP, Bearman G, Edmond MB (2008). Screening for MRSA: a flawed hospital infection control intervention. Infection Control & Hospital Epidemiology.

[CR77] Wertheim HFL, Vos MC, Boelens HAM, Voss A, Vandenbroucke-Grauls CMJE, Meester MHM, Verbrugh HA (2004). Low prevalence of methicillin-resistant *Staphylococcus aureus* (MRSA) at hospital admission in the Netherlands: The value of search and destroy and restrictive antibiotic use. Journal of Hospital Infection.

[CR78] WHO (World Health Organization). 2015. Global action plan on antimicrobial resistance. https://www.who.int/antimicrobial-resistance/global-action-plan/en/. Accessed 28 June 2019.

[CR79] WHO (World Health Organization). 2017. Guidelines for the prevention and control of carbapenem-resistant *Enterobacteriaceae*, *Acinetobacter baumannii* and *Pseudomonas aeruginosa* in health care facilities. World Health Organization Guidelines. 29630191

[CR80] Wiklund S, Fagerberg I, Örtqvist Å, Broliden K, Tammelin A (2015). Staff experiences of caring for patients with extended-spectrum β-lactamase–producing bacteria: a qualitative study. American Journal of Infection Control.

[CR81] Wiklund S, Hallberg U, Kahlmeter G, Tammelin A (2013). Living with extended-spectrum β-lactamase: a qualitative study of patient experiences. American Journal of Infection Control.

[CR82] Wilson APR, Livermore DM, Otter JA, Warren RE, Jenks P, Enoch DA, Tanner G (2016). Prevention and control of multi-drug-resistant Gram-negative bacteria: Recommendations from a Joint Working Party. Journal of Hospital Infection.

[CR83] Woerther PL, Andremont A, Kantele A (2017). Travel-acquired ESBL-producing Enterobacteriaceae: Impact of colonization at individual and community level. Journal of Travel Medicine.

[CR84] Woerther PL, Burdet C, Chachaty E, Andremont A (2013). Trends in human fecal carriage of extended-spectrum β-lactamases in the community: Toward the globalization of CTX-M. Clinical Microbiology Reviews.

